# The mitochondrial genome of *Paragyrodactylus variegatus* (Platyhelminthes: Monogenea): differences in major non-coding region and gene order compared to *Gyrodactylus*

**DOI:** 10.1186/1756-3305-7-377

**Published:** 2014-08-17

**Authors:** Fei Ye, Stanley D King, David K Cone, Ping You

**Affiliations:** Co-Innovation Center for Qinba regions’ sustainable development, College of Life Science, Shaanxi Normal University, Xi’an, 710062 China; Department of Biology, Dalhousie University, Halifax, Nova Scotia B3H 4 J1 Canada; Department of Biology, Saint Mary’s University, Halifax, Nova Scotia B3H 3C3 Canada

**Keywords:** *Paragyrodactylus variegatus*, Mitochondrial genome, *Gyrodactylus*, Gyrodactylidae, Monogenea, *Homatula variegata*, China

## Abstract

**Background:**

*Paragyrodactylus* Gvosdev and Martechov, 1953, a viviparous genus of ectoparasite within the Gyrodactylidae, contains three nominal species all of which infect Asian river loaches. The group is suspected to be a basal lineage within *Gyrodactylus* Nordmann, 1832 *sensu lato* although this remains unclear. Further molecular study, beyond characterization of the standard Internal Transcribed Spacer region, is needed to clarify the evolutionary relationships within the family and the placement of this genus.

**Methods:**

The mitochondrial genome of *Paragyrodactylus variegatus* You, King, Ye and Cone, 2014 was amplified in six parts from a single worm, sequenced using primer walking, annotated and analyzed using bioinformatic tools.

**Results:**

The mitochondrial genome of *P. variegatus* is 14,517 bp, containing 12 protein-coding genes (PCGs), 22 transfer RNA (tRNA) genes, two ribosomal RNA (rRNA) genes and a major non-coding region (NCR). The overall A + T content of the mitochondrial genome is 76.3%, which is higher than all reported mitochondrial genomes of monogeneans. All of the 22 tRNAs have the typical cloverleaf secondary structure, except tRNA^Cys^, tRNA^Ser1^ and tRNA^Ser2^ that lack the dihydrouridine (DHU) arm. There are six domains (domain III is absent) and three domains in the inferred secondary structures of the large ribosomal subunit (rrnL) and small ribosomal subunit (rrnS), respectively. The NCR includes six 40 bp tandem repeat units and has the double identical poly-T stretches, stem-loop structure and some surrounding structure elements. The gene order (tRNA^Gln^, tRNA^Met^ and NCR) differs in arrangement compared to the mitochondrial genomes reported from *Gyrodactylus* spp.

**Conclusion:**

The Duplication and Random Loss Model and Recombination Model together are the most plausible explanations for the variation in gene order. Both morphological characters and characteristics of the mitochondrial genome support *Paragyrodactylus* as a distinct genus from *Gyrodactylus*. Considering their specific distribution and known hosts, we believe that *Paragyrodactylus* is a relict freshwater lineage of viviparous monogenean isolated in the high plateaus of central Asia on closely related river loaches.

## Background

Gyrodactylids are widespread parasites of freshwater and marine fishes, typically inhabiting the skin and gills of their hosts. Their direct life-cycle and hyperviviparous method of reproduction facilitates rapid population growth. Some species are pathogenic to their host (e.g. *Gyrodactylus salaris* Malmberg, 1957) [1] and capable of causing high host mortality resulting in serious ecological and economical consequences [2]. Over twenty genera and 400 species of gyrodactylids have been described [3], most of them being identified by comparative morphology of the opisthaptoral hard parts. This traditional approach for identification of gyrodactylids gives limited information for detailed phylogenetic analysis. Recently, the nuclear ribosomal DNA (rDNA) and the internal transcribed spacers (ITS) of rDNA have been incorporated into the molecular taxonomy of the group [4, 5]. In addition, mitochondrial markers (COI and COII) are also confirmed to be DNA barcoding for *Gyrodactylus* Nordmann, 1832 [6, 7]. But more polymorphic molecular markers suitable for different taxonomic categories are still needed for studying the taxonomy and phylogeny of these parasites.

*Paragyrodactylus* Gvosdev and Martechov, 1953 is a genus of Gyrodactylidae comprising three nominal species, *Paragyrodactylus iliensis* Gvosdev and Martechov, 1953 (=*P. dogieli* Osmanov, 1965), *Paragyrodactylus barbatuli* Ergens, 1970 and *Paragyrodactylus variegatus* You, King, Ye and Cone, 2014, all of which infect river loaches (Nemacheilidae) inhabiting streams in central Asia [8]. The relationship between *Paragyrodactylus* and *Gyrodactylus* has been recently explored. Kritsky and Boeger reported the two genera had a close relationship based on morphological characters [9]. Bakke *et al.* believed the complexity of the attachment apparatus separates *Paragyrodactylus* from *Gyrodactylus* and pondered whether these differences were fundamental or a local diversification within *Gyrodactylus* [3]. Furthermore, You *et al.,* using morphology and molecular data, presented the hypothesis that *Paragyrodactylus* was a relict freshwater lineage of viviparous monogeneans isolated in the high plateaus of central Asia on river loaches [8]. The ambiguous relationship between *Paragyrodactylus* and *Gyrodactylus* emphasizes the need for further molecular study of these genera.

Due to its higher rate of base substitution, maternal inheritance, evolutionary conserved gene products and low recombination [10, 11], mitochondrial genomes provide powerful markers for phylogenetic analysis, biological identification and population studies. In addition, mitochondrial genomes can provide genome-level characters such as gene order for deep-level phylogenetic analysis [12, 13]. To date, the complete mitochondrial DNA sequences of only nine monogeneans are available, including three species of *Gyrodactylus*.

In the present study, the first mitochondrial genome for *Paragyrodactylus*, *P. variegatus*, is sequenced and characterized. We report on its genome organization, base composition, gene order, codon usage, ribosomal and transfer RNA gene features and major non-coding region. Additionally, we provide a preliminary comparison of the gene arrangement within both *Paragyrodactylus* and *Gyrodactylus*.

## Methods

### Specimen collection and DNA extraction

Specimens of *P. variegatus* were collected from the skin and fins of wild *Homatula variegata* (Dabry de Thiersant, 1874) in the Qinling Mountain region of central China. Upon capture the specimens were immediately preserved in 99% ethanol and stored at 4°C. The DNA from one parasite was extracted using a TIANamp Micro DNA Kit (Tiangen Biotech, Beijing, China) according to the manufacturer’s protocol.

### PCR and sequencing

The complete mitochondrial genome of *P. variegatus* was amplified in six parts using a combination of existing primers and newly developed primers generated by primer walking (primers listed in Table 1). For short fragments (<2 kb), PCR reactions were performed in a total volume of 25 μl, containing 3.0 mM MgCl_2_, 10 mM Tris–HCl (pH 8.3), 50 mM KCl, 0.25 mM of each dNTP , 1.25 U rTaq polymerase (TaKaRa, Dalian, China), 0.4 μM of each primer, 45 ng gDNA. Cycling conditions were: an initial denaturation for 1 min at 93°C, followed by 40 cycles of 10 sec at 92°C, 1.5 min at 52–54°C, 2 min at 60°C, and final extension of 6 min at 72°C. For long fragments (>2 kb), the 25 μl PCR reaction consisted of 2.5 mM MgCl_2_, 2.5 μl 10 × LA PCR Buffer II (Mg^2+^ free), 0.4 mM of each dNTP, 1.25 U LA Taq polymerase (TaKaRa, Dalian, China), 0.4 μM of each primer, 45 ng gDNA. Cycling conditions were: an initial denaturation for 1 min at 94°C, followed by 40 cycles of 20 sec at 93°C, 30 sec at 53–54°C, 4–7 min at 68°C, and final extension of 10 min at 68°C. All PCR products were purified with a PCR Purification Kit (Sangon Biotech, Shanghai, China) and sequenced using multiple primers including those which generated the PCR product and new internal primers developed by primer walking.

**Table 1:** **List of PCR primer combinations used to amplify the mitochondrial genome of** ***Paragyrodactylus variegatus***

Primer name	Gene	Sequence(5′ – 3′)	Source
1 F(UND1F)*	ND1	CGHAAGGGNCCNAAHAAGGT	Huyse et al. (2007) [17]
1R*	COI	TAAACTTCTGGATGWCCAAAAAAT	This study
2 F(UNAD5F)	ND5	TTRGARGCNATGCGBGCHCC	Huyse et al. (2007) [17]
2R	COIII	YCARCCTGAGCGAATTCARGCKGG	This study
3 F(U12SF)*	rrnS	CAGTGCCAGCAKYYGCGGTTA	Huyse et al. (2007) [17]
3R(UNAD5R)*	ND5	GGWGCMCGCATNGCYTCYAA	Huyse et al. (2007) [17]
4 F	ND5	ATGTGATTTTTAGAGTTATGCTT	This study
4R(6RNAD5)	ND5	AGGHTCTCTAACTTGGAAAGWTAGTAT	Huyse et al. (2008) [24]
5 F*	COIII	TCTTCWRTTACAGYAACDTCCTA	This study
5R*	ND1	AAACCTCATACCTAACTGCG	This study
6 F*	COI	CTCCTTTATCTGGTGCTCTGGG	This study
6R*	rrnS	GACGGGCGGTATGTACCTCTCT	This study
F236	COIII	TTGTTTTTGATTCCGTGA	This study
F930	CYTB	TTATCTTTGTGGTTCGTTCG	This study
F1568	CYTB	AGGTCAAAGATAGGTGGGTTAG	This study
F2174	ND4	TATAGGAATTTTACCATTATTTA	This study
F2855	ND4	CATGGCTTATCAGTTTG	This study
F3302	tRNA^Gln^	GGTAGCATAGGAGGTAAGGTTC	This study
F8330	COI	TTTAGCGGGTATTTCAAGTA	This study
F8920	COI	GTATTATTCACTATAGGAGGGGTA	This study
R4662	ATP6	ACGAAATAATAAAAATATAAAAAGT	This study
R5283	ND2	TCCAGAAACTAACAATAAAGCAC	This study
R6003	tRNA^Val^	ACCTAATGCTTGTAATG	This study
R6599	ND1	AAACCTCATACCTAACTGCG	This study
R7212	tRNA^Pro^	GCAGCCCTATCAGTAAGACC	This study
R7941	COI	ACCAAGCCCTACAAAACCTG	This study
R10014	rrnL	TCCCCATTCAGACAATCCTC	This study
R10652	rrnS	GCTGGCACTGTGACTTATCCTA	This study
R11375	COII	ATTGTAGGTAAAAAGGTTCAC	This study
R12090	ND6	AAAAAGACAATAAGACCCACTA	This study
R12752	tRNA^Leu(UUR)^	AACACTTTGTATTTGACGCT	This study
R14014	ND5	AGGTTCAAGTAATGGTAGGTCT	This study

*The PCR primers for the long PCR fragment (>2 kb).

### Sequence analysis

Contiguous sequence fragments were assembled using SeqMan (DNAStar) and Staden Package v1.7.0 [14]. Protein-coding (PCGs) and ribosomal RNA (rRNA) genes were initially identified using BLAST (Basic Local Alignment Search Tool) searches on GenBank, then by alignment with the published mitochondrial genomes of *Gyrodactylus derjavinoides* Malmberg, Collins, Cunningham and Jalali, 2007 (GenBank no. EU293891), *G. salaris* (GenBank no. DQ988931) and *Gyrodactylus thymalli* Zitnan, 1960 (GenBank no. EF527269). The secondary structure of the two rRNA genes was determined mainly by comparison with the published rRNA secondary structures of *Dugesia japonica* Ichikawa and Kawakatsu, 1964 (GenBank no. NC_016439) [15]. Protein-coding regions were translated with the echinoderm mitochondrial genetic code. The program tRNAscan-SE v1.21 (http://lowelab.ucsc.edu/tRNAscan-SE/) was used to identify transfer RNA (tRNA) genes and their structures [16], using the mito/chloroplast codon and setting the cove cutoff score to one. The tRNAs, which were not detected by tRNA scan-SE v1.21, were identified by comparing the sequence to *Gyrodactylus* [17, 18]. Tandem Repeat Finder v4.07 was used to identify tandem repeats in non-coding regions [19]. The base composition, codon usage and genetic distance were calculated with MEGA v5.1 [20]. The nonsynonymous (Ka)/synonymous (Ks) values were estimated by the KaKs_Calculator v1.2 with the MA method [21].

## Results

### Genome organization, base composition and gene order

**Figure 1:**
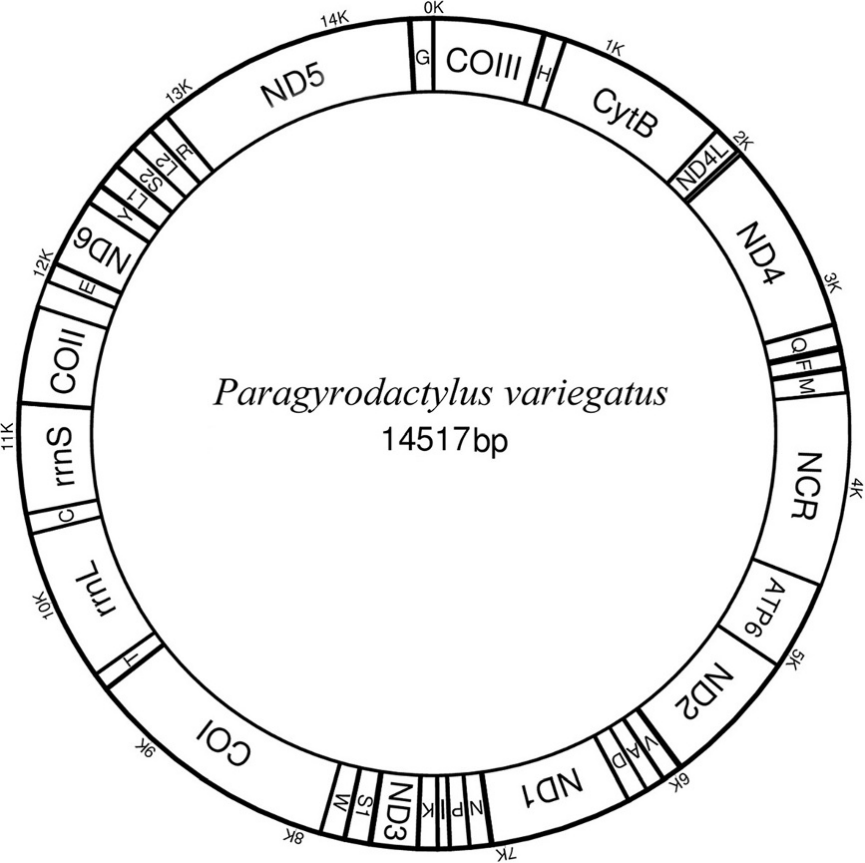
**The gene map for the mitochondrial genome of** ***Paragyrodactylus variegatus*** **.**

The circular mitochondrial genome of *P. variegatus* is 14,517 bp in size (GenBank no. KM067269) and contains 12 PCGs, 22 tRNAs, two rRNA and a single major non-coding region (NCR) (Figure 1). It lacks the ATP8 gene, and all the genes are transcribed from the same strand. The overall nucleotide composition is: T (45.8%), C (9.5%), A (30.4%), G (14.2%), with an overall A + T content of 76.3% (Table 2).

**Table 2:** **Base composition of the mitochondrial genome of** ***Paragyrodactylus variegatus***

Genes	A%	T%	C%	G%	A + T%
Complete genome	30.4	45.8	9.5	14.2	76.3
Protein genes	27.8	47.9	9.5	14.8	75.7
rRNA genes	34.4	40.7	10.6	14.2	75.1
tRNA genes	32.6	40.8	10.8	15.8	73.5
Major non-coding region (NCR)	42.5	40.9	7.2	9.4	83.4

The arrangement of rRNA and protein coding genes of *P. variegatus* is typical for gyrodactylids. However, the gene order of some tRNA genes is different: there are three tRNAs (tRNA^Gln^, tRNA^Phe^, tRNA^Met^) between ND4 and the major non-coding region and five tRNAs (tRNA^Tyr^, tRNA^Leu1^, tRNA^Ser2^, tRNA^Leu2^, tRNA^Arg^) between ND6 and ND5 in *P. variegatus*, while *Gyrodactylus* spp. have one tRNA (tRNA^Phe^) and seven tRNAs (tRNA^Tyr^, tRNA^Leu1^, tRNA^Gln^, tRNA^Met^, tRNA^Ser2^, tRNA^Leu2^, tRNA^Arg^) in the same location, respectively.

### Protein coding genes and codon usage

The total length of all 12 PCGs is 9,990 bp. The average A + T content of PCGs is 75.7% (Table 2), ranging from 70.9% (COI) to 82.9% (ND2). ATG is the typical start codon, except for ND1 and COII, which begins with GTG and TTG, respectively (Table 3). All PCGs terminate with the stop codons TAA, while ND5 uses the codon TAG. The incomplete stop codons were not observed in *P. variegatus*.

**Table 3:** **The organization of the mitochondrial genome of** ***Paragyrodactylus variegatus***

Gene	Position		Size (bp)	Codon		Anticodon	Intergenic nucleotides
	Form	To		Start	Stop		
COIII	1	639	639	ATG	TAA		/
tRNA-His (H)	651	713	63			GTG	11
CYTB	719	1798	1080	ATG	TAA		5
ND4L	1803	2057	255	ATG	TAA		4
ND4	2030	3238	1209	ATG	TAA		-28
tRNA-Gln (Q)	3245	3311	67			TTG	6
tRNA-Phe (F)	3331	3397	67			GAA	19
tRNA-Met (M)	3410	3476	67			CAT	12
NCR	3477	4569	1093				0
ATP6	4570	5082	513	ATG	TAA		0
ND2	5084	5959	876	ATG	TAA		1
tRNA-Val (V)	5974	6040	67			TAC	14
tRNA-Ala (A)	6047	6112	66			TGC	6
tRNA-Asp (D)	6114	6178	65			GTC	1
ND1	6183	7073	891	GTG	TAA		4
tRNA-Asn (N)	7087	7155	69			GTT	13
tRNA-Pro (P)	7159	7221	63			TGG	3
tRNA-Ile (I)	7216	7283	68			GAT	-6
tRNA-Lys (K)	7288	7352	65			CTT	4
ND3	7361	7711	351	ATG	TAA		8
tRNA-Ser^(AGN)^(S1)	7726	7782	57			TCT	14
tRNA-Trp (W)	7792	7858	67			TCA	9
COI	7862	9409	1548	ATG	TAA		3
tRNA-Thr (T)	9418	9484	67			TGT	8
rrnL(16S)	9484	10443	960				-1
tRNA-Cys (C)	10444	10503	60			GCA	0
rrnS (12S)	10505	11216	712				1
COII	11223	11804	582	TTG	TAA		6
tRNA-Glu (E)	11955	12018	64			TTC	150
ND6	12025	12501	477	ATG	TAA		6
tRNA-Tyr (Y)	12507	12573	67			GTA	5
tRNA-Leu^(CUN)^(L1)	12585	12650	66			TAG	11
tRNA-Ser^(UCN)^(S2)	12657	12716	60			TGA	6
tRNA-Leu^(UUR)^(L2)	12719	12788	70			TAA	2
tRNA-Arg (R)	12794	12860	67			TCG	5
ND5	12865	14433	1569	ATG	TAG		4
tRNA-Gly (G)	14446	14513	68			TCC	12

The codon usage and relative synonymous codon usage (RSCU) values are summarized (Table 4). The most frequent amino acids in the PCGs of *P. variegatus* are as follows: Leucine (16.43%), Phenylalanine (13.23%), Serine (12.48%), and Isoleucine (10.67%). The frequency of Glutamine is especially low (0.69%). The codons TTA (Leucine; 12.09%) and TTT (Phenylalanine; 11.48%) are the most frequently used codons. For the third position of the fourfold degenerate amino acid, codons ending with T are the most frequent.

**Table 4:** **Codon usage for the 12 mitochondrial proteins of** ***Paragyrodactylus variegatus***

Codon(AA)	N	%	RSCU	Codon(AA)	N	%	RSCU
UUU(F)	381	11.48	1.74	UAU(Y)	180	5.42	1.72
UUC(F)	58	1.75	0.26	UAC(Y)	29	0.87	0.28
UUA(L)	401	12.09	4.41	UAA(*)	0	0.00	0
UUG(L)	39	1.18	0.43	UAG(*)	0	0.00	0
CUU(L)	68	2.05	0.75	CAU(H)	45	1.36	1.7
CUC(L)	7	0.21	0.08	CAC(H)	8	0.24	0.3
CUA(L)	27	0.81	0.3	CAA(Q)	14	0.42	1.22
CUG(L)	3	0.09	0.03	CAG(Q)	9	0.27	0.78
AUU(I)	175	5.27	1.48	AAU(N)	103	3.10	1.67
AUC(I)	11	0.33	0.09	AAC(N)	18	0.54	0.29
AUA(I)	168	5.06	1.42	AAA(N)	64	1.93	1.04
AUG(M)	68	2.05	1	AAG(K)	48	1.45	1
GUU(V)	150	4.52	2.4	GAU(D)	54	1.63	1.59
GUC(V)	8	0.24	0.13	GAC(D)	14	0.42	0.41
GUA(V)	81	2.44	1.3	GAA(E)	37	1.12	1.32
GUG(V)	11	0.33	0.18	GAG(E)	19	0.57	0.68
UCU(S)	114	3.44	2.2	UGU(C)	65	1.96	1.83
UCC(S)	9	0.27	0.17	UGC(C)	6	0.18	0.17
UCA(S)	65	1.96	1.26	UGA(W)	58	1.75	1.55
UCG(S)	3	0.09	0.06	UGG(W)	17	0.51	0.45
CCU(P)	38	1.15	2.03	CGU(R)	33	0.99	3
CCC(P)	2	0.06	0.11	CGC(R)	4	0.12	0.36
CCA(P)	34	1.02	1.81	CGA(R)	5	0.15	0.45
CCG(P)	1	0.03	0.05	CGG(R)	2	0.06	0.18
ACU(T)	58	1.75	2.37	AGU(S)	104	3.13	2.01
ACC(T)	9	0.27	0.37	AGC(S)	12	0.36	0.23
ACA(T)	30	0.90	1.22	AGA(S)	81	2.44	1.57
ACG(T)	1	0.03	0.04	AGG(S)	26	0.78	0.5
GCU(A)	33	0.99	1.97	GGU(G)	90	2.71	2.05
GCC(A)	7	0.21	0.42	GGC(G)	18	0.54	0.41
GCA(A)	25	0.75	1.49	GGA(G)	46	1.39	1.05
GCG(A)	2	0.06	0.12	GGG(G)	22	0.66	0.5

A total of 3318 codons for *P. variegatus* were analyzed, excluding the stop codons. AA, amino acid; N, number of used codon; % = N/3318; RSCU, relative synonymous codon usage.

### Ribosomal and transfer RNA genes

**Figure 2:**
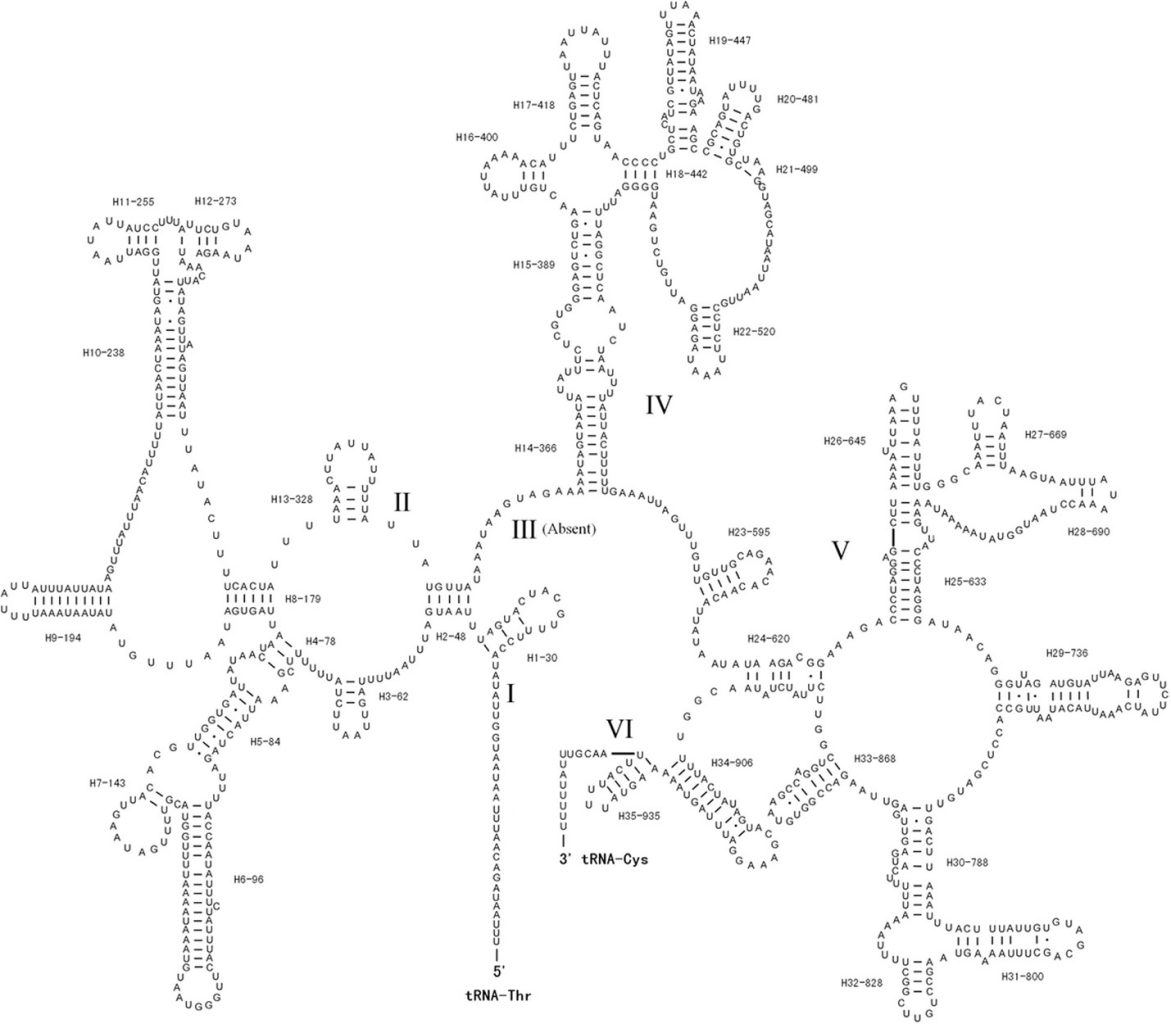
**Inferred secondary structure of the mitochondrial rrnL gene for** ***Paragyrodactylus variegatus*** **.**

**Figure 3:**
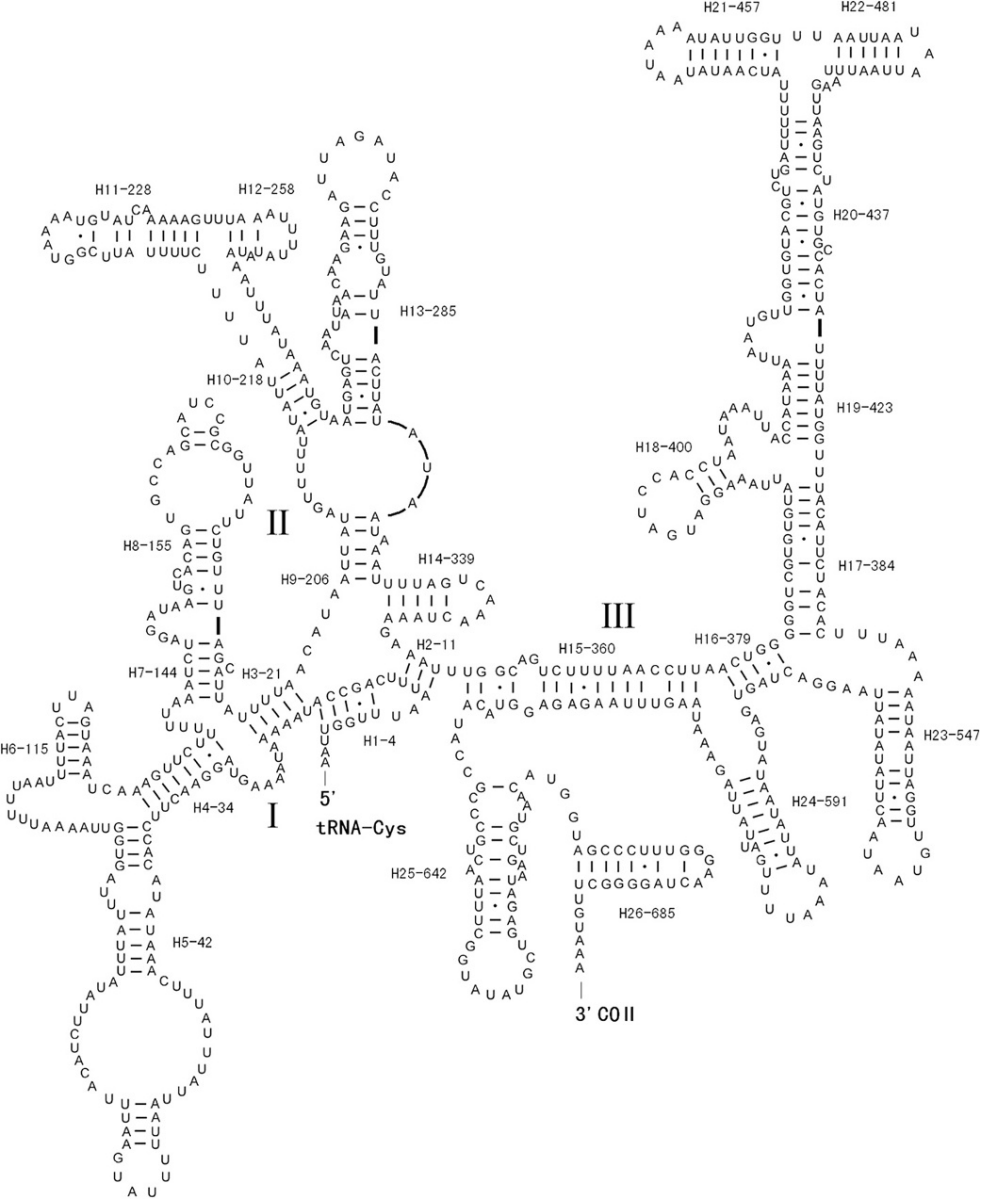
**Inferred secondary structure of the mitochondrial rrnS gene for** ***Paragyrodactylus variegatus*** **.**

The length of large ribosomal subunit (rrnL) and small ribosomal subunit (rrnS) genes of *P. variegatus* are 960 bp and 712 bp, respectively (Table 3). The A + T contents of the rrnL and rrnS of *P. variegatus* are 75.0% and 75.3%, respectively. The predicted secondary structure of rrnL and rrnS of *P. variegatus* is shown in Figure 2 and Figure 3. The secondary structures of these regions contain six and three structural domains, respectively. But domain I of the rrnL lacks a large region at the 5′ end gene, and the domain III is absent in the secondary structure of rrnL of *P. variegatus*.

The 22 tRNA genes of *P. variegatus* vary in length from 57 to 70 nucleotides. Sequences of tRNA^Ile^ and tRNA^Thr^ genes overlap with neighboring genes (Table 3). All of the 22 tRNAs have the typical cloverleaf secondary structure, except for tRNA^Cys^, tRNA^Ser1^ and tRNA^Ser2^ in which each have unpaired dihydrouridine (DHU) arm.

### Synonymous and nonsynonymous substitutions and genetic distance

**Figure 4:**
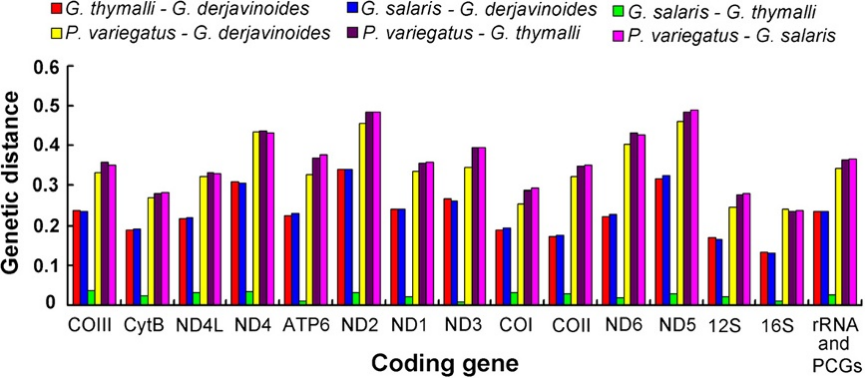
**The genetic distance of protein and rRNA genes of** ***Paragyrodactylus variegatus*** **and** ***Gyrodactylus*** **spp.**

The Ka/Ks values for all 12 PCGs of *P. variegatus* versus *Gyrodactylus* spp. are presented, which all are less than 0.3. The highest average Ka/Ks value is ND2 (0.29), while the Ka/Ks ratios of half the PCGs are low (Ka/Ks < 0.1). The genetic distance between *P. variegatus* and the three reported species of *Gyrodactylus* (*G. thymalli*, *G. salaris* and *G. derjavinoides*) are much greater than among the three species of *Gyrodactylus* (Figure 4). The maximum divergence occurs in ND5 gene (48.9%) between *P. variegatus* and *G. salaris*. In addition, the genetic distances of rRNA genes are lower than protein genes (Figure 4).

### Non-coding regions

**Figure 5:**
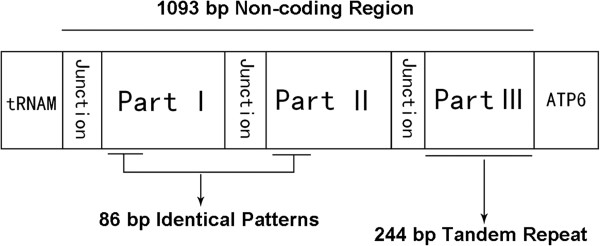
**Organization of the mitochondrial major non-coding region of** ***Paragyrodactylus variegatus*** **.**

**Figure 6:**
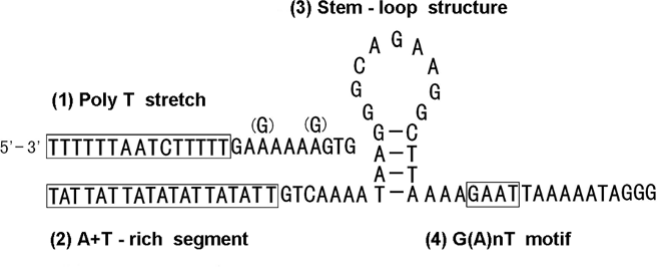
**Predicted structural elements for the mitochondrial major non-coding region of** ***Paragyrodactylus variegatus*** **.** ‘(G)’ is the variation in the identical pattern of part II.

The major non-coding region is 1,093 bp in size, which is highly enriched in AT (83.4%). This non-coding region can be subdivided into six parts including three junctions by the sequence pattern (Figure 5). The sequence of part I and part II is homologous with 81.7% sequence identity. Part III contains six identical repeat units of 40 bp sequence with some sequence modifications: one substitution at the fifth position (the initial repeat unit), three substitutions at the 223rd, 227th and 237th positions and two insertions at the 222nd and 225th positions (the terminal repeat unit). The repeat unit of part III was able to fold into a stem-loop secondary structure. Some predicted structural elements were also found in the sequence of part I and II (Figure 6). In addition, 30 short non-coding regions, all < 151 bp, occur in the mitochondrial genome of *P. variegatus* (Table 3).

## Discussion

### Characteristics of the mitochondrial genome

The mitochondrial genome of *P. variegatus* is 222 bp shorter than that of *G. derjavinoides*, but well within the length range of parasitic flatworms [22, 23]. Differing number and length of the major non-coding region is the main factor that contributes to this difference in genome size. The overall A + T content of *P. variegatus* is higher than that of all reported mitochondrial genomes of monogeneans. The average Ka/Ks values of genes encoding 3 subunits of cytochrome c oxidase and the cytochrome b subunit of cytochrome bc1 complex are lower than genes encoding subunits of the NADH dehydrogenase complex (with the exception of ND1), especially COI and Cytb genes. This feature demonstrates COI, COII, COIII and Cytb genes are more strongly effected by purifying selection pressure compared to subunits of the NADH dehydrogenase genes (except ND1), which is similar to the findings of Huyse *et al*. [24] for *Gyrodactylus derjavinoides*. The degree of functional constraints might be a reason for corresponding degree sequence variations of protein genes. The low Ka/Ks values and genetic distance of COI and Cytb genes also imply that both genes could be used as a useful marker for analyses at higher taxonomic levels. Although sizes of rrnL and rrnS are very similar among *Gyrodactylus* spp. and *P. variegatus,* the sequence similarities are not high. These discrepancies may reflect the variable helices or loops that exist in the rRNA structure.

### The major non-coding region

The mitochondrial genome of *P. variegatus* includes one major non-coding region, which has been frequently observed in other invertebrates. It contains a high A + T content and tandem repeat sequences which could not be found in large non-coding regions (>500 bp) of the published mitochondrial genomes of monopisthocotyleans. We found that length and number of tandem repeat units are similar to those observed in *Microcotyle sebastis* Goto, 1894 [25] *,* contradicting the study of Zhang *et al.* [26] that reported the length and number of repeated motifs were different in the mitochondrial non-coding regions of monopisthocotylids and polyopisthocotylids.

A non-coding region with high A + T content and pertinent elements usually corresponds to the control region for replication and transcription initiation. In the major non-coding region of *P. variegatus*, we found identical patterns within part I and part II. The patterns have only two nucleotide modifications with 2.3% sequence discrepancy; however, the overall difference between the whole sequence of part I and part II is 18.3%. The highly conserved part of the non-coding region is believed to have a functional role. The patterns contain poly-T stretches, a stem-loop structure and some surrounding structure elements (A + T-rich segment and G[A]nT) (Figure 6) which are typical of control regions in insects [27–30]. Although typical control regions are not readily identifiable within the mitochondrial genome of flatworms [17], the predicted secondary structure, conserved element, repeat sequences and high A + T content of major non-coding region in *P. variegatus* implies that this region might play an important role in the initiation of replication and transcription.

In addition, through alignment of non-coding regions sequences between *Gyrodactylus* spp. and *P. variegatus*, we found some conserved motifs in each species with the overall similarity among them being 72.1%. The conserved motifs (>5 bp) mainly existed in the A + T-rich segment and G + A-rich segment. However, whether or not the conserved motifs are present in other species of Gyrodactylidae needs to be assessed with a broader taxon sample.

### Gene arrangements and possible evolutionary mechanisms

**Figure 7:**
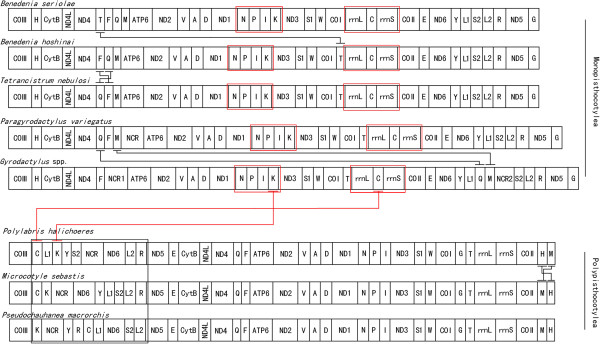
**Gene arrangements of ten monogenean species.** Gene and genome size are not to scale. All genes are transcribed in the same direction (form left to right). Red and black box shows the conserved gene cluster and gene rearrangement hot spot, respectively. The non-coding region (>500 bp) is denoted by the NCR. The same gene arrangement of three *Gyrodactylus* species (*G. salaris, G. derjavinoides* and *G. thymalli*) is shown as *Gyrodactylus* spp.

Five available mitochondrial gene arrangements of monopisthocotylids are shown in Figure 7. The arrangement of all rRNA and protein coding genes are identical throughout all samples, however, the tRNA genes differ in arrangement showing some translocation, particularly long-range translocation. No notable rearrangement hot spot could be found in gene arrangements of monopisthocotylids, however, the major change of gene arrangement among polyopisthocotylids is limited in the COIII-ND5 junction as a gene rearrangement hot spot [26]. Two gene clusters (tRNA^Asn^-tRNA^Pro^-tRNA^Ile^-tRNA^Lys^ and rrnL-tRNA^Cys^-rrnS) were found to be conserved in all mitochondrial genomes of monopisthocotyleans. Nevertheless, the tRNA^Lys^ and tRNA^Cys^ were found in the gene rearrangement hot spot of polyopisthocotyleans. The conserved gene clusters could potentially be a marker used to help define the Polyopisthocotylea and Monopisthocotylea within the monogenea, as well as providing information for a deeper understanding of the evolution of monogenean mitochondrial genomes.

**Figure 8:**
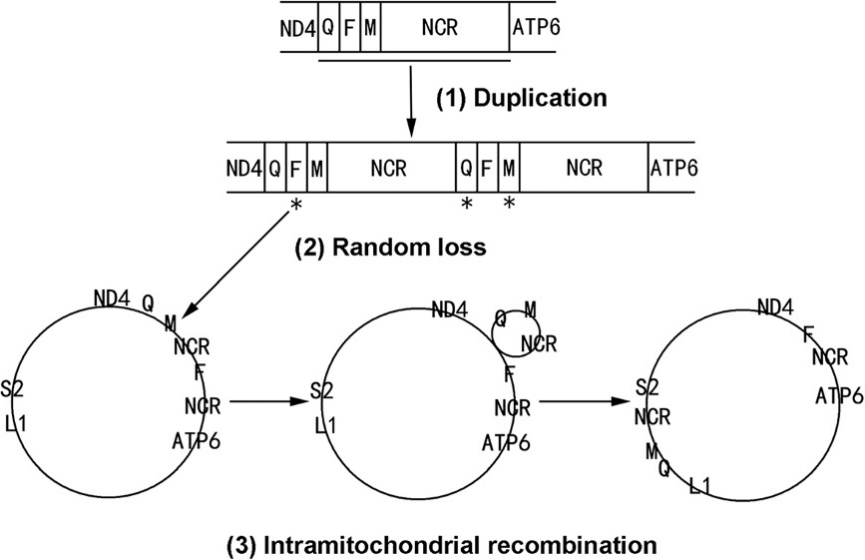
**Possible mechanism of mitochondrial gene rearrangements occurring in** ***Paragyrodactylus variegatus*** **and** ***Gyrodactylus*** **spp.**

Gene rearrangement can be mainly explained by three mechanisms: Duplication and Random Loss Model [31, 32], Duplication and Nonrandom Loss Model [33] and Recombination Model [34]. The variation (tRNA^Gln^, tRNA^Met^ and NCR) of mitochondrial gene order occurring between *P. variegatus* and *Gyrodactylus* spp. could be explained by the duplication and random loss model and recombination model together with the parsimonious scenario. We assume that the process contains three steps: one tandem duplication, random loss, followed by intramitochondrial recombination (Figure 8). We prefer this mechanism for the following reasons: the duplicate NCRs in the mitochondrial genomes of most metazoans can be explained by the duplication and random loss model, but the stepwise mechanism described above is more appropriate to interpret the duplicated NCRs and long-range translocation, meanwhile the rest of the genes remain in their original state. Furthermore, there are several examples of mitochondrial recombination in animals [35–38], and a similar mechanism accounts for the gene rearrangement of other metazoans [39, 40]. In addition, the tRNA^Met^ genes of *Gyrodactylus* spp. are clearly homologous to the tRNA^Met^ gene of *P. variegatus* with 80.6% sequence similarity. However, the tRNA^Gln^ region does have low sequence similarity (66.2%) between the mitochondrial genomes of *Gyrodactylus* spp. and *P. variegatus*, so we cannot be certain that the translocation event happened. As more mitochondrial genomes of gyrodactylids become available, all of the above hypotheses should be tested with respect to gene orders.

## Conclusions

The characteristics of the mitochondrial genome of *P. variegatus* are notably different from *Gyrodactylus* spp., including the gene order, which is similar to other monopisthocotylids. The overall average genetic distance between *Paragyrodactylus* and *Gyrodactylus* based on the rRNA and 12 protein coding genes is remarkably greater than within *Gyrodactylus*. All of these features support *Paragyrodactylus* as a distinct genus. Considering their specific distribution and hosts, we tend towards the view of You *et al.* [8] that *Paragyrodactylus* is a relict freshwater lineage of viviparous monogenean isolated in the high plateaus of central Asia on closely related river loaches.
